# Validation of Inertial Sensor to Measure Barbell Kinematics across a Spectrum of Loading Conditions

**DOI:** 10.3390/sports8070093

**Published:** 2020-06-29

**Authors:** John C. Abbott, John P. Wagle, Kimitake Sato, Keith Painter, Thaddeus J. Light, Michael H. Stone

**Affiliations:** Sport, Exercise, Recreation and Kinesiology, Clemmer College, East Tennessee State University, 1276 Gilbreath Dr, Johnson City, TN 37614, USA; waglej@etsu.edu (J.P.W.); Satok1@etsu.edu (K.S.); zkbp3@etsu.edu (K.P.); lightTj@etsu.edu (T.J.L.); stonem@etsu.edu (M.H.S.)

**Keywords:** resistance training, velocity-based training, athlete monitoring, back squat

## Abstract

The aim of this study was to evaluate the level of agreement in measuring back squat kinematics between an inertial measurement unit (IMU) and a 3D motion capture system (3DMOCAP). Kinematic variables included concentric peak velocity (CPV), concentric mean velocity (CMV), eccentric peak velocity (EPV), eccentric mean velocity (EMV), mean propulsive velocity (MPV), and POP-100: a proprietary variable. Sixteen resistance-trained males performed an incrementally loaded one repetition maximum (1RM) squat protocol. A series of Pearson correlations, 2 × 4 RM ANOVA, Cohen’s *d* effect size differences, coefficient of variation (CV), and standard error of the estimate (SEE) were calculated. A large relationship existed for all variables between devices (*r* = 0.78–0.95). Between-device agreement for CPV worsened beyond 60% 1RM. The remaining variables were in agreement between devices with trivial effect size differences and similar CV magnitudes. These results support the use of the IMU, regardless of relative intensity, when measuring EMV, EPV, MPV, and POP-100. However, practitioners should carefully select kinematic variables of interest when using the present IMU device for velocity-based training (VBT), as certain measurements (e.g., CMV, CPV) do not possess practically acceptable reliability or accuracy. Finally, the IMU device exhibited considerable practical data collection concerns, as one participant was completely excluded and 13% of the remaining attempts displayed obvious internal error.

## 1. Introduction

Athlete monitoring has become an integral component of high-performance athletic development programs and includes both fatigue management (e.g., training loads, training intensity, or questionnaire responses) and the evaluation of program efficacy (e.g., quantification and analysis of physiological responses, performance outcomes) [1,2]. Monitoring barbell velocity has gained popularity and provides an example of within-session data collection to guide the training process—especially in managing fatigue through the consideration of acute velocity loss and in targeting specific motor qualities, though the latter is the topic of considerable debate [3,4]. Nonetheless, velocity measures are a potentially efficacious monitoring augmentation for resistance training programs [1,2]. It has been suggested that unlike other methods such as ratings of perceived exertion (RPE) and repetitions in reserve (RIR), barbell velocities provide near instantaneous quantitative feedback, which may assist in guiding load selection, which is valuable for proper workload management [3,5]. This feedback—though most frequently used to provide data to help manage training stress and quality—may also be used to provide the athlete external motivation that drives greater movement intent [6]. Though an ancillary benefit of having access to the technology, this may be one of the more valuable components considering the favorable strength and power adaptations that movement intent drives [7,8]. 

These benefits, among others, drove the emergence of affordable and practical barbell kinematic measuring devices to provide this feedback readily within sessions [9]. In general, the types of devices can be divided into three primary categories: (1) camera-based, (2) linear position transducers (LPT), and (3) inertial measurement units (IMUs). The use of camera-based systems is least likely to be utilized in a practical setting due to the prerequisite of space, personnel requirements, and monetary investment. Linear position transducers have been the long-standing practical preference due to high inter-device agreement with video analysis [10,11,12] and relative ease of use. Caution has been advised when considering LPT as a criterion measure due to limitations including the ability to match segment references including trajectories [13]. The inability to match segment references likely degrades inter-device agreement, which does not necessarily represent device superiority. 

Inertial measurement units, newer to the market relative to video and LPT, may prove to be the most practically accessible tool due to cost and ease of use. The integration of an accelerometer, gyroscope, and magnetometer allows for measurement and tracking in three dimensions [14]. These devices rely on wireless transmission of raw data and proprietary algorithms to derive kinematic measures. The quality of the data is typically contingent upon several factors including IMU orientation and placement, complexity of movement, inter- and intra-individual technique differences, and sampling frequency [15,16]. Due to these potential mediating influences on data quality, it is important that there is a continued exploration of the reliability and validity of IMUs against the laboratory-grade measurement devices, especially as the commercial availability increases. The purpose of this study was to investigate the validity of a commercially available wireless IMU and the respective algorithms used to derive a variety of phasic kinematic variables against a 3D motion capture system (3DMOCAP). The authors hypothesized that the commercially available IMU and its respective algorithms would provide similar measurements relative to the gold-standard 3DMOCAP for phase-specific kinematic variables. However, the authors also hypothesized that the strength of this agreement would decrease as load progressed to higher relative intensities.

## 2. Materials and Methods

### 2.1. Experimental Approach to the Problem

Subjects completed all testing during a single session, which began with a general dynamic warm-up. To compare the IMU and 3DMOCAP, resistance-trained males performed a one repetition maximum (1RM) squat test protocol, beginning at 20% of self-reported 1RM and progressing in 5–10% increments until technical or actual failure occurred. During testing, barbell kinematic data were simultaneously recorded using 3DMOCAP (4 cameras, Vicon System, Nexus 1.8.5, Vicon Motion Systems, Oxford, UK) and a wireless triaxial IMU (Bar Sensei, Assess2Perform, Steamboat Springs, CO, USA) (Figure S1). 

One participant displayed a particularly ballistic transition from the eccentric (ECC) to the concentric (CON) phase and was removed from the analysis. In addition, a total of 30 attempts (13%) were removed due to IMU internal errors (i.e., miscalculations) and as a result 226 attempts were used for analysis. The IMU internal errors were recognized as estimated ECC displacements below 0.25 m and/or POP-100 results less than 0.001. 

### 2.2. Subjects

Sixteen resistance-trained males (age: 25.9 ± 5.2 years, training age: 6.0 ± 4.5 years, height: 177.1 ± 6.1 cm, body mass: 91.4 ± 10.2 kg, back squat 1RM/Body Weights: 1.7 ± 0.3) participated in the current investigation. Participants were informed and familiarized with the study and its protocols. Participants signed written informed consent approved by the university’s Institutional Review Board and had no current or prior injuries that would interfere with the completion of the protocol.

### 2.3. Procedures

Subjects were allotted 5 min for a self-selected warm-up, inclusive of dynamic stretches and several unloaded barbell back squat repetitions before beginning the 1RM squat protocol. Self-selected warm-ups were used to accommodate the different preferences of subjects, who were all experienced in resistance training and consequently equipped to properly prepare themselves for the first experimental load condition. Furthermore, permitting self-selected warm-ups improves the ecological validity of the study. However, the authors did control for the duration (5 min) and absolute squat intensity (unloaded, 20 kg barbell) permitted in the warm-up for standardization purposes. The protocol consisted of two repetitions at each 10% load increment between 20 and 70% of self-reported 1RM and one repetition at each 5% load increment from 75% 1RM until technical or actual failure. Rest duration between sets was a self-selected amount of time between 3 and 5 min [17,18]. Successful squat performance criteria included a squat depth that allowed the hip to descend below the patella [19], and the ability to complete the repetition; all repetitions were assessed by two certified investigators. All successful repetitions across the spectrum of relative loads were considered for subsequent validation analysis. Barbell kinematics were simultaneously recorded with 3DMOCAP and wireless triaxial IMU, both sampling at 100 Hz. The sampling rate of 3DMOCAP was set to match the frequency reported by the company for the wireless IMU. The 3DMOCAP tracked the barbell using a single reflective marker centered on the end of the barbell [20,21]. The IMU was placed outside the center knurling of the barbell with the logo facing upward, as is the recommended placement according to the company. 

### 2.4. Data Processing

The 3DMOCAP was processed and the following variables were identified: eccentric peak velocity (EPV), eccentric mean velocity (EMV), concentric peak velocity (CPV), concentric mean velocity (CMV), mean propulsive velocity (MPV), and POP-100. Eccentric peak velocity was the highest instantaneous velocity value obtained between the initiation of downward movement and the maximal downward displacement. Eccentric mean velocity was the arithmetic mean velocity between the initiation of downward movement and the maximal displacement. Concentric peak velocity was the highest instantaneous velocity value obtained between the initiation of upward movement and maximal upper displacement. Concentric mean velocity was the arithmetic mean velocity between the initiation of upward movement and maximal upper displacement [22]. Mean propulsive velocity was the arithmetic mean velocity between the start of the concentric movement until the acceleration of the bar is lower than gravity [23]. POP-100, a proprietary variable of the company and not a traditional kinematic variable, was the instantaneous barbell velocity occurring 100 ms after the initiation of the concentric phase. To account for and diminish noise, a low-pass fourth-order Butterworth filter with a 15 Hz cutoff frequency was applied to the 3DMOCAP data [16]. The cutoff frequency was determined using residual analysis during pilot testing. Barbell kinematics recorded by the barbell accelerometer were exported from the company’s smart-phone application (Assess2Perform, Steamboat Springs, CO, USA). 

### 2.5. Statistical Analyses

Descriptive statistics including mean and standard deviation (SD) were calculated for all variables per relative intensity. A series of 2 × 4 RM ANOVA tests were completed for each variable to determine differences between the two devices on all data. Data were grouped into 4 bandwidths, 20–39%, 40–59%, 60–69%, 70–100%, for RM ANOVA analysis to evaluate the effect of the device, intensity, and potential interaction effects. Normal distribution was assessed with a Shapiro–Wilk test. No violations of normality of distribution were observed; in cases of violation of sphericity a Greenhouse–Geisser correction was applied. The alpha level was set as *p* ≤ 0.05. Pearson’s *r* values were calculated and interpreted with magnitude thresholds of 0–0.1, 0.1–0.3, 0.3–0.5, 0.5–0.7, 0.7–0.9, and 0.9–1.0 as trivial, small, moderate, large, very large, and nearly perfect. Cohen’s *d* effect size differences were also calculated relative to percent 1RM and interpreted with magnitude thresholds of 0–0.20, 0.21–0.60, 0.61–1.20, 1.21–2.0, and 2.0 and above as trivial, small, moderate, large, and very large [24]. Within- and between-device agreement was assessed for each variable. Within-device variations relative to percent 1RM were assessed using coefficients of variation (CV). Between-device agreement was assessed using standard error of the estimate (SEE), relative to each percent 1RM, and Bland–Altman analysis, both relative to the devices overall.

## 3. Results

**Figure 1:**
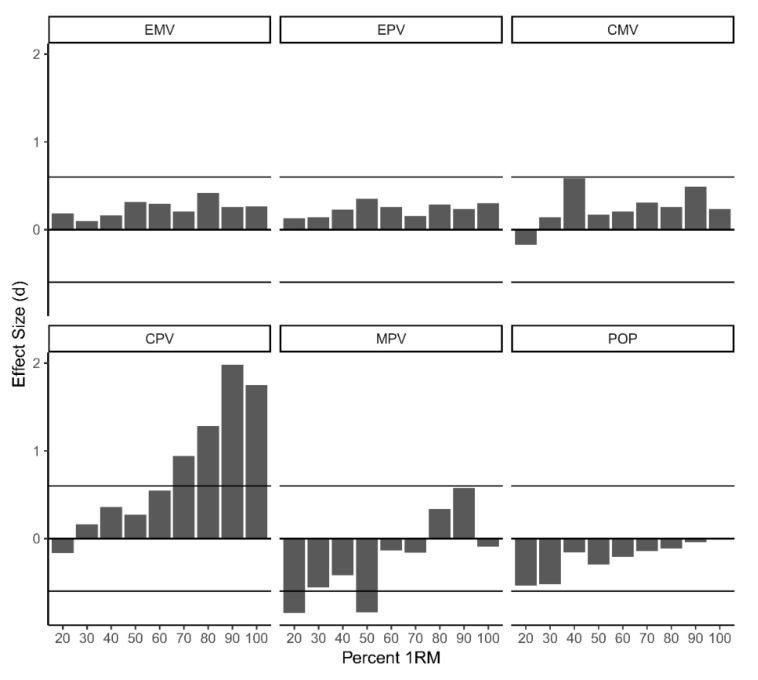
Magnitude-based inferences, Cohens *d*, for EMV, EPV, CMV, CPV, MPV, POP-100. EMV = eccentric mean velocity; EPV = eccentric peak velocity; CPV = concentric peak velocity; CMV = concentric mean velocity; MPV = mean propulsive velocity. Limits set at 0.6, representing a large effect size difference.

A series of 2 × 4 repeated-measures ANOVA tests indicated no significant differences between devices or subjects for EMV, EPV, CMV, and POP-100. MPV measurements were significantly affected by the interaction of device (F(3,72) = 4.36, *p* < 0.01) and intensity with no between-subject effect; Bonferroni post hoc analysis revealed no significant difference between devices, *p* = 0.23. CPV displayed significant interaction (F(3,72) = 12.55, *p* < 0.01, and between-subject effects F(1,24) = 10.36, *p* < 0.01. Bonferroni post hoc analysis revealed significant differences between devices, *p* < 0.01. Simple main effects revealed a significant difference for CPV between devices above at higher intensities (40–69: F(1) = 11.02, *p* < 0.01; 70–100: F(1) = 25.76, *p* < 0.01, Table 1). Magnitude-based inferences revealed small differences across the spectrum of intensities for EMV, EPV, CMV, and POP-100. Small differences were observed for CPV for intensities less than 60%, *d* = −0.16–0.55 and for MPV above 60%, *d* = −0.16–0.57 (Figure 1).

**Figure 2:**
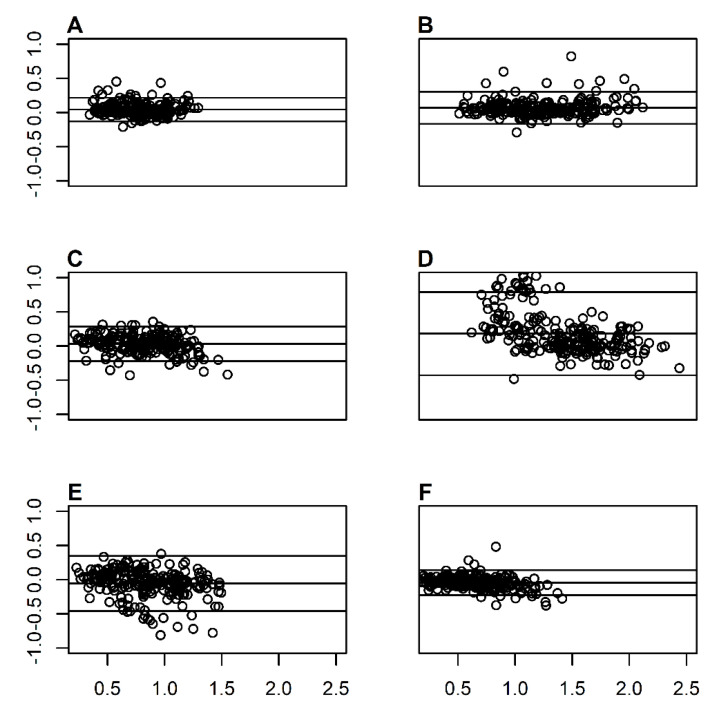
Bland–Altman plots displaying means (x-axis) and differences (y-axis) for 3D motion capture and IMU data for eccentric mean velocity (**A**), eccentric peak velocity (**B**), concentric mean velocity (**C**), concentric peak velocity (**D**), mean propulsive velocity (**E**), POP-100 (**F**).

**Figure 3:**
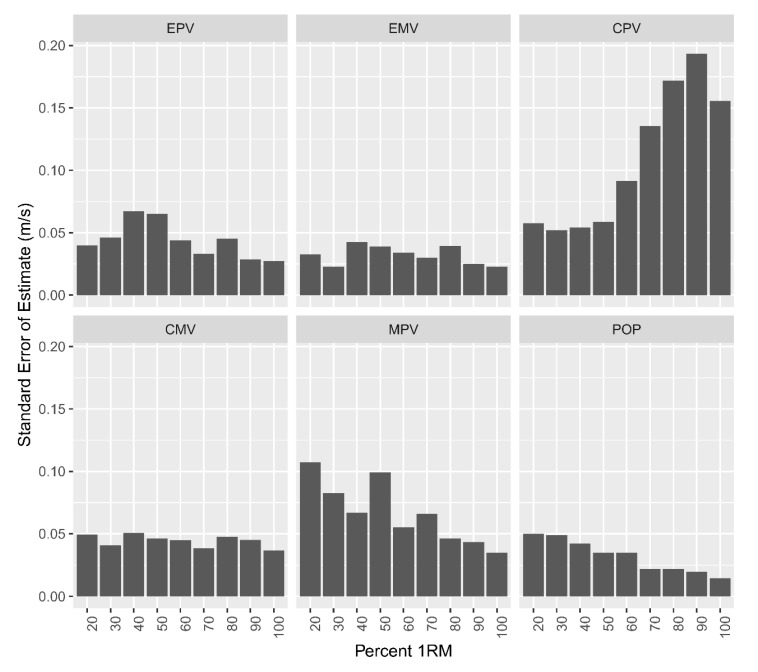
Standard error of the estimate per variable, intensity, and device. IMU = inertial measurement unit; 3DMOCAP = Vicon 3D motion capture analysis; EMV = eccentric mean velocity; EPV = eccentric peak velocity; CPV = concentric peak velocity; CMV = concentric mean velocity; MPV = mean propulsive velocity.

When considering all intensities, a nearly perfect relationship existed between devices for EMV, EPV, CMV, POP-100, and MPV, *r* = 0.92, *r* = 0.95, *r* = 0.90, *r* = 0.95, *r* = 0.93, respectively. A very strong relationship existed between devices for CPV (*r* = 0.78). Bland–Altman analysis indicated bias toward 3DMOCAP at slower velocities for CPV, meaning 3DMOCAP tended to provide higher CPV measurements. Conversely, the Bland–Altman analysis indicated bias toward IMU at faster velocities for MPV and POP-100 (Figure 2). The magnitudes of coefficients of variation were similar across the spectrum of intensities for EMV, EPV, and POP-100 (Table 2). Dissimilarly, the magnitude of CV for CMV and CPV increased at a greater rate in the IMU device, particularly at higher intensities. SEE revealed a substantial larger error above 60% 1 RM for CPV (Figure 3). Notably, MPV, POP-100, and EPV displayed reduced error with increasing intensity (Figure 3). 

## 4. Discussion

The purpose of this study was to investigate the validity of a commercially available wireless IMU and respective algorithms used to calculate a variety of phasic kinematic variables. The results of this investigation indicate that, overall, the IMU provides similar kinematic measurement values relative to the 3D motion capture system, except for CPV. The IMU device also exhibited less than 100% collection success, as one participant was completely excluded and 13% of the remaining attempts displayed obvious internal error. Overall, the two devices were in agreement and with consistent bias (i.e., higher measurement values using 3D motion capture) when considering ECC characteristics. When examining CON characteristics, a strong relationship was observed between all variables except CPV. However, when considering the Bland–Altman analysis of CPV, there was a bias present across the spectrum of relative intensities, with the IMU generally producing greater kinematic measurement values at higher relative intensities and the opposite being true at lower relative intensities. 

During the current investigation, the two measurement devices shared a nearly perfect relationship and trivial-to-small effect differences in CMV across all intensities. This is partly in agreement with Banyard and colleagues [22] under a similar design but comparing CMV measurements of an IMU and LPT device instead. This group also reported a progressively increasing CV in CMV measured by an IMU device at higher intensities; the magnitudes of CV reported are similar to the findings of the current study [22]. However, the effect size difference between the IMU and the laboratory measurement values was notably larger at higher intensities compared to the findings of the current investigation, which could be in part due to increased sensitivity of the equipment used in the laboratory comparison method [22]. Mitter and colleagues [13] were able to demonstrate reasonable agreement between 3DMOCAP and an IMU device at a more similar sampling rate of 200 Hz. However, the authors did acknowledge that movement cadence (e.g., the inclusion of an isometric pause between the ECC and CON phases) influenced the IMU device accuracy. It is also worth noting that the previous studies [13,22] discussed IMU devices that were mounted on the subject’s forearm, whereas the current investigation used an IMU mounted on the barbell [13,22]. 

In the current study, between-device agreement was weakest when considering CPV, although still practically interpreted as a large relationship. Previous work [25] found nearly perfect agreement (*r* = 0.91) between an IMU and LPT when evaluating a spectrum of five fixed incremental loads that were estimated to correspond to 25–85% back squat 1RM. The results of the current investigation demonstrated greater variation between devices when using intensities above 60% 1RM. The greater relationship between devices reported by Balsalobre-Fernandez and colleagues [25] relative to the current investigation could partly be due to a less extensive use of higher relative intensities, which would logically reduce the variation observed. The relationship could also have been artificially inflated due to the use of a Smith machine in this investigation, which also minimized variability of movement in the sagittal plane. 

Unlike CPV, MPV appeared to be in closer agreement to 3DMOCAP at higher intestines, as represented by trivial-to-small effect differences and SEE ranging from 0.03 to 0.06 m/s using intensities greater than 60% 1RM. However, a lone exception and noticeable increase in variation occurred at 100% 1RM. This increase in MPV variation may be a function of training at 100% 1RM and not of the device or even exercise selection, as the same increase in variation was previously observed when measuring the bench press using a LPT [23]. Carroll and colleagues [26] reported increased variation in barbell back squat concentric mean velocity as loads incrementally progressed from 61 to 100% 1RM, with the greatest variation at a failed 1RM attempt (CV = 55%). Considering this, the ideal scenario includes similar magnitudes of CV within the IMU and 3DMOCAP that increase with higher relative intensities. Though this was observed in the current investigation regarding some metrics (e.g., EMV, EPV, POP-100), the CV of CPV measured with the IMU became markedly larger compared to the CV of 3DMOCAP when using higher intensities. 

Because CPV and CMV are both influenced by the sticking point and the other metrics are not, it is reasonable to infer that the embedded algorithm of the IMU was unable to account for unique kinematic aspects of the sticking region, ultimately driving the decreased agreement in measurement relative to 3DMOCAP. This is most easily demonstrated using POP-100, which captures the velocity achieved at 100 ms of the CON phase. The sticking region occurs later than 100 ms into the CON phase of the back squat [27], leaving the IMU algorithm’s calculation of POP-100 theoretically unaffected by the kinematic variability introduced thereafter. Supporting this notion, POP-100, different than CPV and CMV, shared a nearly perfect relationship, trivial-to-small effect size differences, and similar magnitude CV across the spectrum of loads comparing the IMU and 3DMOCAP. The degradation of the observed relationships when considering CON variables that occur after the sticking region may be due to a combination of factors including, but not limited to, kinematic variations within and between subjects during the sticking region of the CON phase, IMU sampling frequency being too slow for certain phase of lift, and lack of integration of a gyroscope to include unusual sagittal plane barbell movement and rotation or the barbell during the back squat. In order to distinguish the relative contributions of these factors to IMU error, raw data from the IMU must be examined, which is beyond the scope of the current investigation.

The degradation of the agreement between devices is more apparent in CPV than in CMV. SEE, CV, and Cohen’s *d* all clearly expose the reduction in agreement between devices for CPV, whereas only CV for CMV was noticeably affected by higher relative intensities. Data aggregation based on relative intensities when calculating SEE and Cohen’s *d* reduces the resolution of interpretation, hence CV is utilized to provide insight relative to the dispersion of the data. CMV as measured by the IMU is more variant than 3DMOCAP though it appears to be in agreement. Thus, if using this device, CMV is an accurate measure but lacks precision with higher relative intensities. 

These results suggest that more refinement is needed in IMU devices and algorithms to accurately identify and analyze CON movements at higher intensities. To the authors’ knowledge, there are no studies that evaluate ECC movement velocities, particularly with the interest of velocity-based training (VBT) technology. The strong agreement between devices for ECC movement provides insight on the possibilities of IMU accuracy when proper precautions are implanted to incorporate barbell movement variation. There were notable limitations of the current investigation that should be considered. First, only the back squat was used for evaluation of the IMU device. Second, the raw IMU data were unavailable, which limited the authors’ inferences as to why differing levels of agreement were present at different intensities. Future investigations should explore this raw data, especially considering the POP-100’s high reliability and apparent precision observed in the current investigation. Velocity magnitude achieved prior to the sticking region can substantially affect sticking region mechanics [28], with lower velocities (i.e., lower POP-100 values) elongating the sticking region itself and increasing the likelihood of repetition failure [28,29]. 

## 5. Conclusions

With the current model of this IMU, practitioners need to be aware that there is an obvious error from the IMU considering the ability to capture measurements and the results of the measurements. The results of the current investigation suggest that measurements of CON peak velocity agree between IMU and 3DMOCAP below 60% 1RM. However, this is a major limitation, as most athletes frequently train above 60% 1RM during strength training. The remaining kinematic variables considered appear to be valid at all progressive load conditions. Most notably, the IMU appears to be able to adequately measure CMV, which is the most frequently used metric of interest to practitioners when prescribing the squat in resistance training programs. As further technological development continues, the CON portion of lifting needs to be the primary focus for improvement. The current study suggests that the ECC portion of the task is reasonably valid as compared to the 3D motion capture system; however, the CON portion must be calculated more precisely. The current study provides evidence that may allow users and engineers to understand the products strengths and weaknesses when considering use or improvement strategies.

## Figures and Tables

**Table 1:** Mean ± standard deviations (SDs) of velocity measures analyzed per percent one repetition maximum (1RM) and per method (inertial measurement unit (IMU) and 3D motion analysis).

Percent 1RM	Device	EMV	EPV	CPV	CMV	MPV	POP-100
20%	IMU	0.91 ± 0.24	1.47 ± 0.38	1.94 ± 0.27	1.18 ± 0.22	1.32 ± 0.2	1.04 ± 0.25
	3DMOCAP	0.96 ± 0.23	1.52 ± 0.39	1.89 ± 0.29	1.15 ± 0.15	1.2 ± 0.17	0.91 ± 0.22
30%	IMU	0.92 ± 0.21	1.48 ± 0.32	1.81 ± 0.22	1.1 ± 0.17	1.26 ± 0.2	0.98 ± 0.22
	3DMOCAP	0.94 ± 0.2	1.53 ± 0.38	1.84 ± 0.2	1.12 ± 0.12	1.17 ± 0.14	0.88 ± 0.18
40%	IMU	0.84 ± 0.17	1.31 ± 0.3	1.62 ± 0.22	0.93 ± 0.16	1.08 ± 0.17	0.8 ± 0.18
	3DMOCAP	0.87 ± 0.21	1.38 ± 0.34	1.69 ± 0.17	1.02 ± 0.11	1.05 ± 0.12	0.77 ± 0.17
50%	IMU	0.8 ± 0.19	1.24 ± 0.32	1.57 ± 0.24	0.92 ± 0.19	1.03 ± 0.18	0.73 ± 0.18
	3DMOCAP	0.85 ± 0.18	1.36 ± 0.36	1.63 ± 0.21	0.94 ± 0.11	0.96 ± 0.13	0.67 ± 0.16
60%	IMU	0.77 ± 0.18	1.21 ± 0.28	1.39 ± 0.25	0.82 ± 0.16	0.9 ± 0.18	0.66 ± 0.18
	3DMOCAP	0.83 ± 0.18	1.28 ± 0.3	1.51 ± 0.2	0.85 ± 0.12	0.87 ± 0.13	0.63 ± 0.17
70%	IMU	0.72 ± 0.18	1.13 ± 0.29	1.13 ± 0.27	0.7 ± 0.13	0.72 ± 0.14	0.57 ± 0.14
	3DMOCAP	0.75 ± 0.16	1.18 ± 0.29	1.36 ± 0.21	0.73 ± 0.1	0.73 ± 0.11	0.55 ± 0.15
80%	IMU	0.6 ± 0.17	0.97 ± 0.3	0.95 ± 0.3	0.6 ± 0.15	0.6 ± 0.15	0.47 ± 0.13
	3DMOCAP	0.67 ± 0.16	1.06 ± 0.29	1.28 ± 0.21	0.63 ± 0.11	0.62 ± 0.11	0.45 ± 0.15
90%	IMU	0.56 ± 0.13	0.87 ± 0.22	0.74 ± 0.25	0.45 ± 0.13	0.45 ± 0.11	0.38 ± 0.13
	3DMOCAP	0.59 ± 0.13	0.92 ± 0.22	1.21 ± 0.23	0.51 ± 0.1	0.49 ± 0.1	0.38 ± 0.15
100%	IMU	0.57 ± 0.14	0.91 ± 0.24	0.63 ± 0.27	0.34 ± 0.15	0.39 ± 0.13	0.37 ± 0.13
	3DMOCAP	0.61 ± 0.15	0.98 ± 0.26	1.08 ± 0.24	0.37 ± 0.09	0.35 ± 0.09	0.36 ± 0.15

IMU = inertial measurement unit; 3DMOCAP = Vicon 3D motion capture analysis; EMV = eccentric mean velocity; EPV = eccentric peak velocity; CPV = concentric peak velocity; CMV = concentric mean velocity; MPV = mean propulsive velocity.

**Table 2:** Coefficients of variation (95% CIs) of velocity measures analyzed per percent 1RM and per method (IMU and 3D motion analysis).

Percent 1RM	Device	EMV	EPV	CPV	CMV	MPV	POP-100
20%	IMU	26.63 (20.41–38.47)	26.03 (19.96–37.54)	14.17 (11.04–20.05)	18.97 (14.64–27.02)	15.42 (12–21.84)	23.7 (18.22–34.03)
	3DMOCAP	23.94 (18.4–34.39)	25.8 (19.79–37.19)	15.53 (12.09–22)	13.09 (10.2–18.49)	18.89 (14.58–26.89)	24.44 (18.78–35.15)
30%	IMU	22.85 (17.66–32.46)	21.83 (16.89–30.95)	12.44 (9.74–17.41)	15.79 (12.34–22.19)	15.73 (12.3–22.1)	22.7 (17.55–32.24)
	3DMOCAP	21.21 (16.41–30.04)	24.82 (19.15–35.39)	10.61 (8.32–14.83)	10.38 (8.14–14.5)	23.57 (18.21–33.53)	20.98 (16.24–29.71)
40%	IMU	19.78 (15.46–27.51)	23.22 (18.1–32.48)	13.39 (10.57–18.47)	17.58 (13.81–24.38)	16.13 (12.71–22.33)	23.08 (17.99–32.27)
	3DMOCAP	24.34 (18.95–34.11)	24.48 (19.07–34.33)	10.28 (8.13–14.15)	10.4 (8.22–14.31)	22.48 (17.53–31.4)	22.52 (17.57–31.47)
50%	IMU	24.04 (18.57–34.24)	26 (20.04–37.16)	15.38 (12.03–21.6)	20.89 (16.17–29.58)	17.98 (13.95–25.34)	25.29 (19.5–36.09)
	3DMOCAP	21.07 (16.31–29.84)	26.15 (20.15–37.39)	12.56 (9.84–17.59)	11.82 (9.27–16.54)	24.47 (18.89–34.87)	23.97 (18.51–34.12)
60%	IMU	22.9 (17.85–32.01)	23.42 (18.25–32.77)	17.86 (13.98–24.77)	19.24 (15.05–26.74)	20.2 (15.79–28.12)	27.35 (21.23–38.55)
	3DMOCAP	21.33 (16.65–29.74)	23.01 (17.94–32.18)	13.34 (10.53–18.41)	13.73 (10.83–18.95)	14.82 (11.69–20.48)	26.5 (20.6–37.3)
70%	IMU	24.88 (19.59–34.18)	25.36 (19.96–34.87)	23.97 (18.89–32.89)	18.82 (14.89–25.63)	19.21 (15.2–26.17)	24.89 (19.6–34.2)
	3DMOCAP	21.71 (17.14–29.67)	24.79 (19.52–34.05)	15.66 (12.48–21.24)	14.27 (11.38–19.32)	22.31 (17.61–30.52)	27.89 (21.9–38.53)
80%	IMU	28.25 (22.09–39.31)	30.75 (23.99–43.01)	31.43 (24.5–44.03)	25.51 (20.01–35.32)	25.71 (20.16–35.61)	28.05 (21.95–39.03)
	3DMOCAP	24.27 (19.05–33.53)	27.9 (21.83–38.79)	16.6 (13.18–22.68)	17.49 (13.88–23.92)	18.92 (14.91–25.92)	33.58 (26.11–47.26)
90%	IMU	22.87 (17.51–33.11)	24.84 (18.98–36.1)	33.36 (25.24–49.47)	29.94 (22.75–44.01)	25.58 (19.53–37.23)	34.88 (26.34–51.94)
	3DMOCAP	21.7 (16.62–31.34)	23.69 (18.12–34.34)	19.19 (14.73–27.59)	19.56 (15.01–28.14)	20.45 (15.68–29.46)	39.7 (29.79–59.97)
100%	IMU	24.1 (17.57–38.56)	26.12 (19–42.02)	43.77 (31.05–75.29)	43.02 (30.56–73.74)	34.59 (24.9–57.24)	35.54 (25.55–59.03)
	3DMOCAP	24.73 (18.02–39.63)	26.42 (19.22–42.54)	22.51 (16.44–35.86)	24.79 (18.06–39.73)	26.16 (19.03–42.08)	42.17 (30–72.01)

IMU = inertial measurement unit; 3DMOCAP = Vicon 3D motion capture analysis; EMV = eccentric mean velocity; EPV = eccentric peak velocity; CPV = concentric peak velocity; CMV = concentric mean velocity; MPV = mean propulsive velocity.

## Supplementary Materials

The following are available online at https://www.mdpi.com/2075-4663/8/7/93/s1, Figure S1: Camera and IMU orientation.
