# Supplementary material for: Validation of Inertial Sensor to Measure Barbell Kinematics across a Spectrum of Loading Conditions
Source: Sports (Basel). 2020 Jun 29;8(7):93. doi: 10.3390/sports8070093 (PMC7404789; doi:10.3390/sports8070093)
Supplement: Supplementary file 1 [file sports-08-00093-s001.pdf]

sports

MDPI

Supplementary Materials: Validation of Inertial Sensor to Measure Barbell Kinematics across a Spectrum of Loading Conditions

John C. Abbott *, John P. Wagle, Kimitake Sato, Keith Painter, Thaddeus J. Light and Michael H. Stone

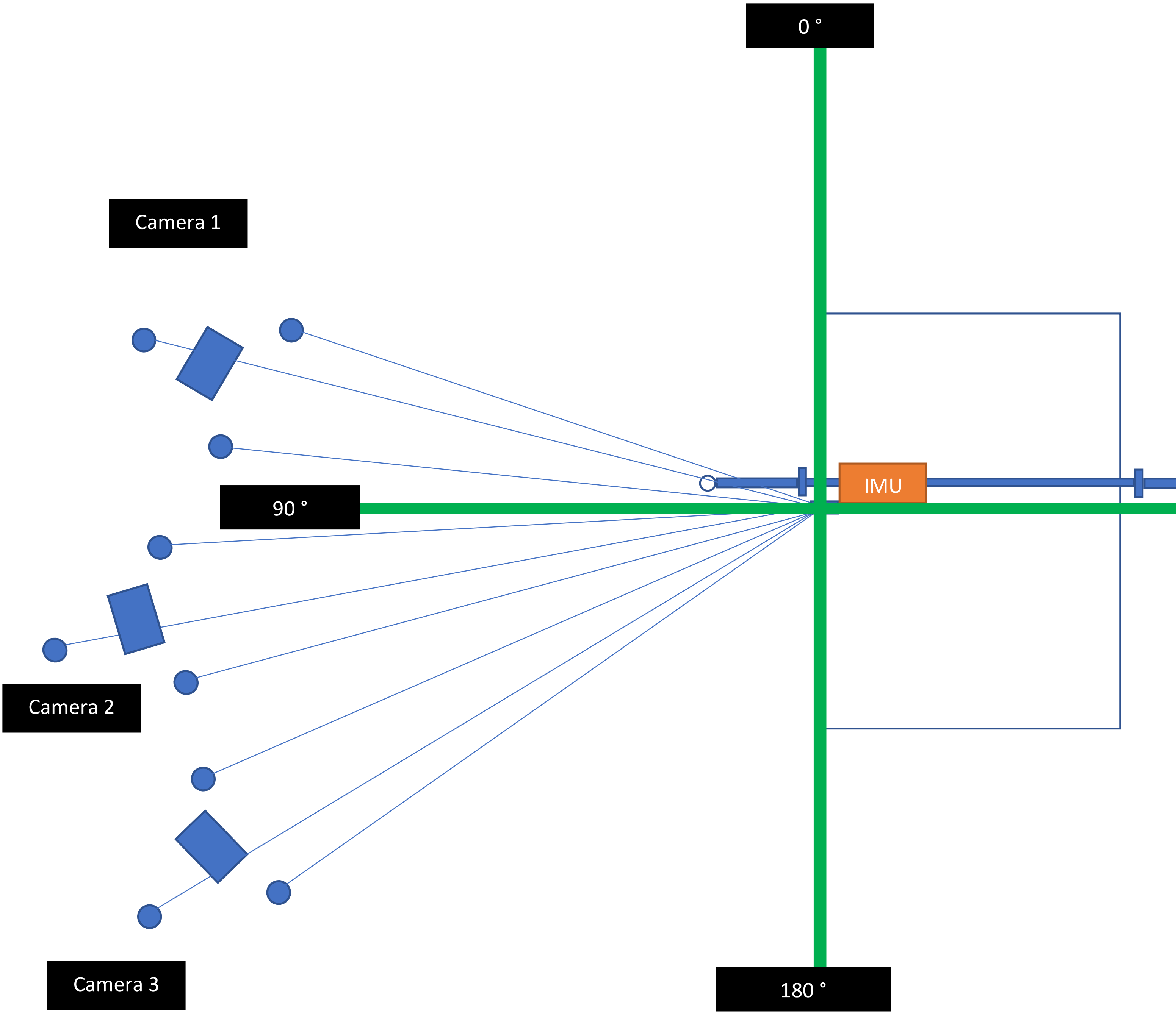

Camera 1 foot placement: 3.96 m at 73 degrees, 4.76 m at 75 degrees, 4.32 m at 82 degrees

Camera 2 foot placement: 4.05 m at 110 degrees, 4.71 m at 100 degrees, 4.98 m at 112 degrees

Camera 3 foot placement: 5.04 m at 125 degrees, 4.62 m at 144 degrees, 5.58 m at 133 degrees

IMU placement on barbell just outside of hand placement, logo facing towards the ceiling

*Diagram not drawn to scale

Figure S1. Camera and IMU orientation.
